# A case report on crossed aphasia in dextrals: Consideration about clinical features and neural network

**DOI:** 10.1097/MD.0000000000017660

**Published:** 2019-10-25

**Authors:** Michele Torrisi, Patrizia Pollicino, Francesco Corallo, Giuliana Vermiglio, Anna Lisa Logiudice, Carmela Mantarro, Cinzia Calabrò, Placido Bramanti, Rocco Salvatore Calabrò, Rosa Morabito, Silvia Marino

**Affiliations:** Istituto di Ricovero e Cura a Carattere Scientifico (IRCCS) Centro Neurolesi Bonino Pulejo, Messina, Italy.

**Keywords:** crossed aphasia, right hemisphere, stroke

## Abstract

**Rationale:**

The term crossed aphasia in dextrals (CAD) describes aphasia following a right hemisphere lesion in right-handed subjects. The diagnostic criteria for CAD, defined on the basis of clinical cases observed over the years, are aphasia; lesion in right hemisphere; strong preference for right hand use without familial history of left handedness; structural integrity of left hemisphere; and absence of brain damage in childhood. The studies of CAD have mainly been focused on the neurobiological mechanisms underlying the functional neurocognitive lateralization and organization of the brain, such as a dissociation between language and handedness, language and praxis, or other cognitive functions.

Patient concerns: We described a case of a patient affected by an aphasic syndrome following cerebral hemorrhage located in right hemisphere.

Diagnosis: Considering the correlation between clinical data and instrumental investigations such as magnetic resonance imaging, we diagnose the patient with non-fluent aphasia. Specifically, the patient came to our attention showing a trans-cortical mixed aphasia that, later, developed in a trans-cortical motor aphasia. Contrary to most cases of CAD, our patient does not show apraxia and visuo-spatial neglect. Interventions language and visual attention when latter functions are related to right hemisphere.

**Interventions:**

The rehabilitation program consisted in exercises stimulating verbal fluency, comprehension, reading, and writing.

**Outcomes:**

After 5 months of rehabilitation patient showed significant improvement in comprehension and absence of echolalia.

**Lessons:**

At present there is no agreement about pathogenesis of CAD and neural mechanism is still unclear. Considering the clinical symptomatology, we can argue that we observed a non-fluent aphasia. However, a more large sample should be studied to asses the role of brain circuits.

## 1 Introduction

Aphasia is one of the most common cognitive disorders resulting from a stroke, with frequencies ranging between 15% and 42% in acute stage and 25% to 50% in rehabilitative settings.^[1,2]^ The patients affected by this syndrome may exhibit inability in expressive language (Broca aphasia, transcortical motor aphasia, global aphasia) or in receptive language (Wernicke aphasia, transcortical sensorial aphasia, conduction aphasia). The first typologies are called “non fluent,” the others “fluent.” Aphasia negatively affects quality of life^[3]^ and functional recovery.^[4,5]^ In addition, a strong correlation with depression is reported especially for the non-fluent type.^[6]^ It is established that this disorder is attributable to lesions of left hemisphere (generally located in fronto, temporal circumvolution, and arcuate fasciculi) consider as dominant for both right and left-handed patients. Nevertheless, an aphasic syndrome resulting from lesions to right hemisphere are possible.

This condition occurs in approximately 30% of left-handed patients but is extremely uncommon in right-handed. Bramwell^[7]^ introduced the term crossed aphasia (CA) to denote any aphasic syndrome resulting from a cerebral lesion “ipsilateral” to the dominant hand. Then, it was found that this phenomenon was rare only for the right-handed and researchers chosen to adopt the term “crossed aphasia in dextral” (CAD) to describe aphasia following a right hemisphere lesion in a right-handed subject.^[8]^ The diagnostic criteria for CAD, defined on the basis of clinical cases observed over the years, are aphasia; lesion in right hemisphere; strong preference for right hand use without familial history of left handedness; structural integrity of the left hemisphere; and absence of brain damage in childhood. Some authors have proposed to exclude patients with illiteracy, bilinguism or ideographic, and tone language because these factors could cause a right-hemisphere lateralization, but this distinction has not received agreement.^[9]^ The comorbidity of CAD and apraxia has been also investigated.^[10]^ Over the years, authors have attempted to focus pathogenesis and lesion sites accounting for CA but theories are controversial. Certainly, it is an extremely rare phenomenon, reporting an average below 3%.^[11,12]^ This percentage does not include non-vascular etiology of CAD.

We described a case of a patient affected by an aphasic syndrome following cerebral hemorrhage located in right hemisphere to assess a correlation with clinical and neuropsychological assessment.

## 2 Case description

**Figure 1:**
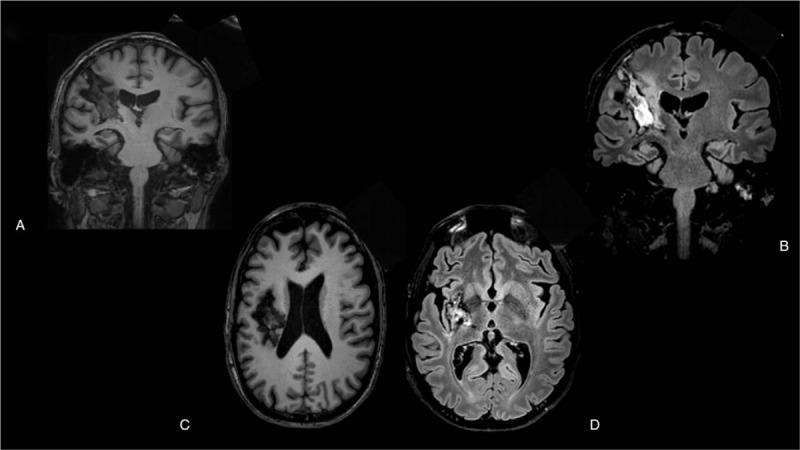
(A) Coronal T1-weighted image. (B) Coronal fluid attenuated inversion recovery (FLAIR)-weighted image. (C) Axial T1-weighted image. (D) Axial FLAIR-weighted image.

A 41-year-old man with a medium level of education, previous affected by Marfan syndrome, chronic renal failure, and sarcoidosis, arrived in our hospital center to undertake neurorehabilitation after a cerebral hemorrhage and consecutive he was craniotomy surgical treatment. Magnetic resonance (MR) results showed a right frontonucleocapsular lesion (see Fig. 1). In the left temporal cortexhemisphere an arachnoid cyst was detected which, however, was not considered decisive in the genesis of the language disorder.

During first days, he showed a severe left hemiplegia and he appeared no responsive to any stimulation, reporting a Coma Recovery Scale's scores of 9 NIHSS of 17. After 1 month, the patient started to execute simple command, show interaction with people and produce a very poor speech, characterized by echolalia and perseveration. After 1 month, considering the patient completely conscious, his speech became more fluid but indicating a clear aphasic syndrome with scarce fluency, agrammatism, paraphasia, comprehension's difficulties, and persistent echolalia. In addition, he showed unawareness about his clinical condition, space–time disorientation, and severe restlessness.

We evaluated the patient by Neuropsychology Exam for Aphasia (ENPA)^[13]^ to investigate language abilities and Edinburgh Handedness Inventory (EHI)^[14]^ to asses hand dominance in daily activities. As illustrated in Table 1, at first evaluation patient reported insufficient scores in all ENPA sub-tests except for word repetition. The most frequent error was semantic in naming sub-test and neologism in reading. The EHI scores, obtained by a caregivers’ interview was +100, evidence of a total right-handedness. The relatives did not report family history of left-handedness. We also evaluated visuo-spatial impairments and apraxia by Spinnler e Tognoni Oral and Ideomotor Apraxia scales and Bells Test observing that patient did not show these difficulties. He showed some difficulties in the execution of tasks on command rather than on imitation, but it was attributable to comprehension abilities not fully preserved. The rehabilitative program, realized twice a day for 5 days a week, included exercise based on stimulation of verbal fluency (i.e., sentences completion, adjective's attribution to noun, listing specimens of a category ecc), reconstruction of decomposed sentences, comprehension proofs (i.e., relation, categorization, and command's execution), reading and writing. After 5 months patient was dismissed. He showed a significant improvement in comprehension abilities. Echolalia has disappeared. On the others language domains there was not significant improvements. Motor and sensory impairment were just a little bit improved (CRS-R: 16; NIHSS: 12).

**Table 1:**
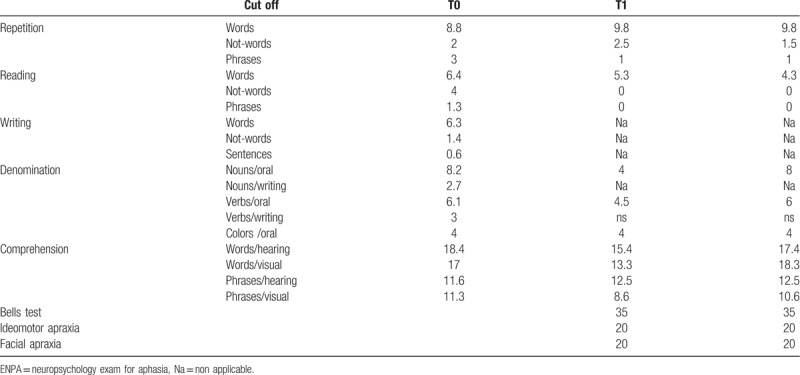
Scores obtained by the patient at the evaluations by ENPA sub-tests, neglect's exam, and apraxia's scales.

## 3 Discussion

We described a patient showing a clear CAD syndrome. His clinical manifestation respected all diagnostic criteria defined over the years. In addition, the criteria of illiteracy and bilingualism that according to some authors could cause a cerebral representation of language more ambivalent, are not present in our case. The first criteria for CAD (aphasia) are quite generic, including all aphasia's sub-types. Some systematic reviews found that Broca's sub-type is the most common.^[8,12]^ Considering the initial presence of echolalia, the preserved repetition and the improvement of comprehension ability led us to consider his syndrome as mixed trans cortical developed a trans-cortical motor. As regard to sex, in all analyzes conducted about CAD, men are more frequently affected than women.^[9]^

Contrary to most cases of CA, our patient does not show apraxia and visuo-spatial neglect. These manifestations are quite common in CA patients, as reported in literature.^[15,16]^ Relating to apraxia, constructional is the most common followed by buccal; limb apraxia is much less frequent.^[17,18]^ Probably, it reveals that oral and constructional praxis tends to lateralize with language skills whereas limb praxis tends to lateralize with hand dominance. Some authors reported a dissociation of language and limb praxis, exemplified by case of crossed aphasia with intact limb praxis and conversely, crossed limb apraxia with intact linguistic functions.^[19]^ This lead to consider the disconnection between language dominance and handedness too, consistent with the interpretation that left hemisphere controls motor skills even if there is an atypical cerebral dominance for language. On the other hand, the percentage of visuo-spatial neglect in CA patients, more common than apraxia, and the frequent co-occurrence with constructional apraxia suggests that at least the visuoperceptual attentional abilities tend to remain in right hemisphere.

In literature poor attention was paid to the cerebral areas damaged in the right hemisphere. In a study conducted on 7 stroke patients satisfying criteria for CAD, a clear involvement of lentiform nucleus, considered by authors as the neural substrate responsible for CAD was found.^[19]^ In a descriptive analysis of 167 cases, Coppens et al^[8]^ did not highlighted correlation between site lesions and aphasia sub-types. We can suppose that frontonucleocapsular area damaged in our patients is the equivalent of left-hemisphere circuits responsible of aphasia. Among the explanations of the pathophysiological mechanism underlying language reversed cerebral dominance in CAD subjects, language functions are not lateralized at subcortical level. As a consequence of an absence of cerebral language dominance at this level, we can assume an overrepresentation of subcortical CAD. On the other hand, we should assume that right hemisphere implied right deep structures as important for language and visual attention when latter functions are both subserved by right hemisphere.

An alternative theory about genesis of CAD was proposed by Sapir et al.^[20]^ They believed that the absence of right shift gene in a small subset of population could predict a random lateralization of cognitive functions, including language. Subcortical crossed aphasia, although rare, have been described in monolinguals following a stroke of the posterior limb of the internal capsule and head of the caudate nucleus, and anterior part of the internal capsule, lentiform nucleus, and head of the caudate. The right parahippocampal gyrus, claustrum, frontal lobe, and precentral gyrus were also locations found to be involved in CAD.^[19]^ At present there is no agreement about pathogenesis of CAD and neural mechanism is still unclear. In details, the role of brain circuits involved should be further investigated. Considering the clinical symptomatology, we can argue that we observed a non-fluent aphasia.^[20,21]^ Specifically, the patient came to our attention showing a trans-cortical mixed aphasia that, later, developed in a trans-cortical motor aphasia.

Our case could suggest additional question about this phenomena which still appears very controversial to date. In particular, it is important to understand how subcortical lesions of right hemisphere could influence language performance in patients with vascular lesions.

## Author contributions

**Conceptualization:** Michele Torrisi

**Data curation:** Giuliana Vermiglio, Patrizia Pollicino; Anna Lisa Logiudice, Francesco Corallo

**Investigation:** Michele Torrisi, Anna Lisa Logiudice, Francesco Corallo, Carmela Mantarro, Cinzia Calabrò

**Resources:** Rosa Morabito, Giuliana Vermiglio

**Supervision:** Silvia Marino, Rocco Salvatore Calabrò, Placido Bramanti.

**Validation:** Silvia Marino,

**Visualization:** Silvia Marino, Placido Bramanti, Rocco Salvatore Calabrò
